# New targets for the treatment of follicular lymphoma

**DOI:** 10.1186/1756-8722-2-50

**Published:** 2009-12-23

**Authors:** Nishant Tageja, Subhash Padheye, Prasad Dandawate, Ayad Al-Katib, Ramzi M Mohammad

**Affiliations:** 1 Department of Internal Medicine, Wayne State University School of Medicine, Detroit, MI- 48201, USA; 2 Drug design and Molecular Medicine Research group, Department of Chemistry, D.Y. Patil University, Pune, India; 3 Department of Internal Medicine, Division of Hematology/Oncology, Wayne State University School of Medicine, Detroit, Michigan, USA

## Abstract

The last two decades have witnessed striking advances in our understanding of the biological factors underlying the development of Follicular lymphoma (FL). Development of newer treatment approaches have improved the outlook for many individuals with these disorders; however, with these advances come new questions. Given the long-term survival of patients with FL, drugs with favourable side-effect profile and minimal long-term risks are desired. FL is incurable with current treatment modalities. It often runs an indolent course with multiple relapses and progressively shorter intervals of remission. The identification of new targets and development of novel targeted therapies is imperative to exploit the biology of FL while inherently preventing relapse and prolonging survival. This review summarizes the growing body of knowledge regarding novel therapeutic targets, enabling the concept of individualized targeted therapy for the treatment of FL.

## Introduction

Non-Hodgkin's Lymphoma (NHL) represents the fifth-leading cause of cancer deaths in the United States and the second-fastest growing cancer in terms of mortality. The incidence rate of NHL has nearly doubled in the last four decades with an annual increase of 4%, due to reasons that are not entirely clear. Approximately 180 Americans are diagnosed with NHL each day [1].

Follicular Lymphoma (FL) is the second most common form of NHL prevailing in the United States [2]. Most patients have a widely spread disease at diagnosis, with involvement of multiple lymph nodes, liver and spleen. Marrow biopsy is positive in 40% of the patients at diagnosis [3]. Despite an advanced stage, the clinical course of disease is usually indolent, with waxing and waning lymphadenopathy over a period of many years. The disease, however, is not curable with available treatment [4,5], and most patients tend to relapse after treatment with shorter intervals of remission in between. In approximately 30% of patients, the disease progresses more rapidly with transformation into Diffuse Large B-Cell Lymphoma (DLBCL) and early death. The molecular biology underlying this phenomenon and the factors associated with the risk of transformation are not entirely known [6].

Incurability of FL with the current treatment, which includes the frontline use of monoclonal antibody to CD20, rituximab (Rituxan, Genentech Inc. and Biogen Idec, USA), leaves a wide-scope for development of future strategies to provide durable complete remissions (CR) and extended quality of life. Given the long-term survival of patients with FL, drugs with favorable side-effect profile and minimal long-term risks are preferred. Recent years have witnessed a marked improvement in our understanding of the biological factors underlying the development of FL. The identification of new targets and development of novel targeted therapies is imperative to exploit the biological indolence of FL while inherently preventing relapse and prolonging survival.

## Apoptotic pathway in follicular lymphoma

The term apoptosis has a Greek origin, meaning 'falling or dropping off', which was coined by Kerr in 1972 to describe the morphological processes leading to programmed cellular self-destruction [7]. It is a tightly regulated and highly efficient pathway of cell death characterized by cell shrinkage, chromatin condensation, and membrane blebbing [8]. At the molecular level, it is a chain of events with positive- and negative-regulatory loops that eventually culminate in the activation of a proteolytic cascade involving members of the caspase family. The process of apoptosis can be divided into initiation and execution phases. Initiation of apoptosis occurs by signals from two alternative convergent pathways: the extrinsic pathway which is receptor mediated, and the intrinsic pathway which is initiated in mitochondria.

The extrinsic pathway involves death receptors, such as type 1-TNF receptor and FAS (CD95). Death receptors bind to their ligands, cross-link, and provide a binding site for an adapter protein with a death domain (FADD). FADD binds an inactive form of caspase-8 and -10 in humans [8]. Multiple procaspase-8 molecules are brought into proximity and cleave one another to generate active enzymes, initiating the execution phase [8,9].

The intrinsic pathway is characterized by the release of pro-apoptotic molecules into the cytoplasm from mitochondria. These molecules belong to the Bcl-2 family of proteins. Bcl-2 and Bcl-X_L _are anti-apoptotic proteins that reside in the mitochondrial membrane, but are replaced by pro-apoptotic molecules when the cell is deprived of survival signals. This leads to an alteration in mitochondrial permeability which releases cytochrome c that binds to Apaf-1 in the cytosol, and this complex activates caspase-9 [10]. Caspases-8 and -9 are initiator caspase enzymes. After an initiator caspase is cleaved to generate its active form, the enzymatic death program is set in motion by rapid and sequential activation of executioner caspases (caspases- 3, -6 and -7) [11].

## A) Bcl-2 inhibitors

*CED-3 *and *CED-4 *were identified as genes essential for programmed cell death (PCD), while *CED-9 *was found to inhibit the process of apoptosis in *C. elegans *[12,13]. Vaux and Adams described the first mammalian homolog of *CED-3 *in 1988 and named it Bcl-2. Bcl-2 transfected B- cells were found to be resistant to apoptosis, normally induced in B-cells by IL-3 withdrawal. Thus, it was demonstrated for the first time that tumorigenesis depends not only on the ability to escape growth control but also on the ability to escape apoptosis [14].

The Bcl-2 gene codes for a 25-kDa protein that resides on the cytoplasmic face of the outer mitochondrial membrane (OMM), nuclear envelope and endoplasmic reticulum (ER). There are a total of 25 genes in the Bcl-2 family known to date. The Bcl-2 and related proteins are a growing family of molecules that share at least one of four homologous regions termed Bcl homology domains (BH1 to BH4). These domains mediate homo- and heterotypic dimer formation amongst Bcl-2 family members [15-18]. Bcl-2 and its similar pro-survival homologs, Bcl-X_L _and Bcl-W, contain all four BH domains. The other pro-survival members contain a minimum of two domains, BH1 and BH2 [19].

Members of this family fall into three main groups based on their structure and function: the anti-apoptotic proteins, which include Bcl-2 and Bcl-X_L_; the pro-apoptotic proteins, which can be further subdivided to include multi-domain proteins, such as Bax and Bak; and lastly, the Bcl homology domain 3 (BH3) only proteins, which includes Bid, Bik, Bim, Bad, Puma and Noxa. The BH3-only proteins are pro-apoptotic and display homology with other family members only in the alpha helical and amphipathic BH3 segments [18,19].

A balance between members of the Bcl-2 family is believed to determine the permeability of the mitochondria and release of proteins that mediate cell death [20]. The pro-survival proteins maintain organelle integrity since Bcl-2 directly or indirectly prevents the release of cytochrome c from mitochondria. In a normal cell, basal levels of pro-survival Bcl-2 like proteins prevent Bax and Bak from becoming activated. Upon transmission of stress signals by the cell, BH3-only proteins become activated and competitively bind to a hydrophobic groove on the anti-apoptotic proteins, thereby neutralizing them. This action displaces Bax and Bak and allows them to form multimers that aggregate on the endoplasmic reticulum (ER) and mitochondrial membranes, triggering a cascade of events leading to cell death [21-23]. A central checkpoint of apoptosis that occurs at the mitochondria is the activation of caspase-9 [24]. The BH4 domain of Bcl-2 and Bcl-X_L _can bind to the C-terminal portion of Apaf-1 and consequently inhibits the association of caspase-9 with Apaf-1[25].

The BH1 and BH2 domains of Bcl-2 family members (Bcl-2, Bcl-X_L _and Bax) show a striking similarity to the overall fold of the pore-forming domains of bacterial toxins. Therefore it has been suggested that Bax- and Bax-like proteins might mediate caspase-independent death via channel-forming activity, which would promote the mitochondrial permeability transition [26]. An inappropriately low rate of apoptosis may prolong the survival or reduce the turnover of abnormal cells. This could facilitate accumulation of chromosomal aberrations, leading to uncontrolled proliferation and tumor initiation.

### Bcl-2 as a Molecular Target

The characteristic cytogenetic alteration in FL is a translocation involving the Bcl-2 gene: t(14;18)(q32;q21). This translocation, which is present in approximately 85% of FL cases, places Bcl-2 under the control of immunoglobulin heavy chain (IgH) enhancer on chromosome 14, resulting in constitutive overexpression of Bcl-2 [27,28]. Thus, de-regulated expression of this gene consequently leads to impaired apoptotic signalling. Consequently transfection of Bcl-2 *in vitro *is capable of increased cell viability and decreased apoptosis of lymphoma cells which additionally confer resistance of these cells to chemotherapeutic drugs [29].

In the recent past, Bcl-2 has been established as a target for improving the treatment of B-cell malignancies using antisense oligodeoxynucleotides to reduce Bcl-2 gene expression [30]. Thus, addition of oblimersen to fludarabine plus cyclophosphamide regime significantly increased the complete and partial response rate (CR, PR) in patients with relapsed or refractory chronic lymphocytic leukaemia (CLL) patients, particularly those that are fludarabine-sensitive, as well as among patients who achieve response during course of their disease [31].

A number of pharmacological approaches have been used to identify Bcl-2 family inhibitors that mimic the actions of the proapoptotic BH3 domains [32]. Structural studies have revealed that BH1, BH2 and BH3 domains in anti-apoptotic proteins fold into a domain containing hydrophobic groove on its surface. As discussed previously, the BH3 domain of BH3 only proteins bind to this groove, thus neutralizing the Bcl2-like proteins [33]. It has been hypothesized that a small-molecule inhibitor (SMI) that binds to this BH3 binding site in Bcl-2 may be capable of blocking the heterodimerization of Bcl-2, leading to aggregation of Bak and Bad.

### Small molecule inhibitors (SMI) of Bcl-2

#### A. Apogossypol (ApoG2)

**Figure 1:**
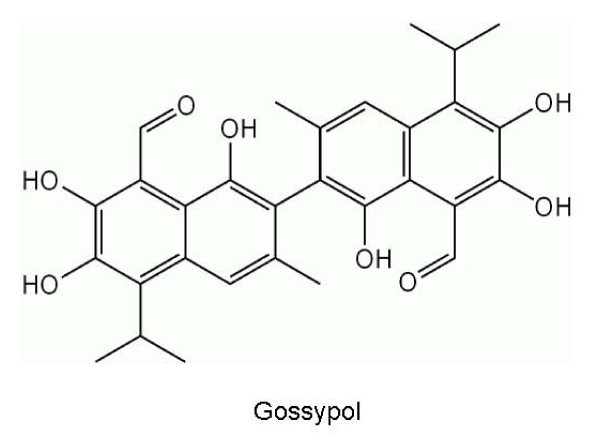
**Chemical structure of Gossypol**.

ApoG2 is a semi-synthetic analog of gossypol that was shown to have modest affinity for Bcl-2, Bcl-X_L _and Mcl-1[34]. Gossypol (Figure 1) is a natural polyphenolic aldehyde that was extracted in its racemic form from cottonseed and extensively investigated as a male contraceptive agent [35]. However, the practical applications of its important properties have been prevented by the toxicity and unpleasant side effects, including emesis and diarrhea. A considerable body of research indicated that the toxicity of gossypol is related to the reactions of the aldehyde groups on the molecule, suggesting that removal of the aldehyde groups from a gossypol molecule could theoretically reduce its toxicity. However, it was unclear if gossypol's biological activity was also tied to the presence of the reactive aldehyde groups. The negative enantiomer of gossypol, AT-101, was found to be clinically active, its use in humans was associated with hepatotoxicity and gastrointestinal (GI) toxicity [36].

**Figure 2:**
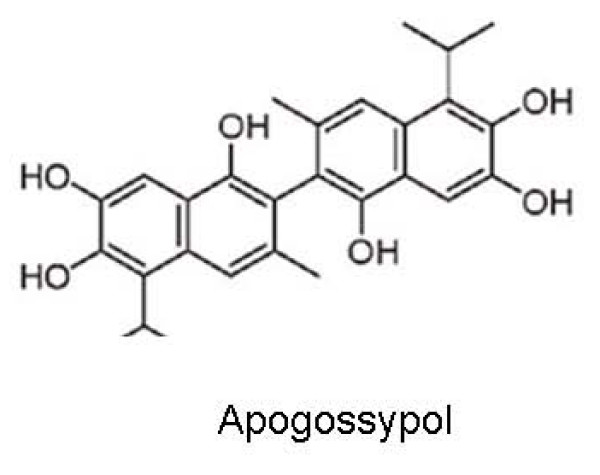
**Chemical Structure of Apogossypol**.

ApoG2 (Figure 2) was developed after eliminating the two reactive aldehydes from gossypol. It has been found to compete with the BH3 peptide-binding sites on Bcl-2, Bcl-X_L_, Mcl-1, Bcl-W, and Bcl-B, but not Bfl-1, with IC_50 _value of 0.5 to 2 μM [36]. Comparison of the *in vitro *activity of gossypol and ApoG2 on the National Cancer Institute (NCI) panel of 59 tumor cell lines has suggested that these compounds have overlapping yet non-identical mechanisms [37]. Our lab has shown that ApoG2 can activate the initiator caspase-9, and the effector caspase-3, and induce caspase cleavage at nanomolar concentrations. In addition, ApoG2 activates PARP and AIF which have been implicated in the final stages of apoptosis. It is likely that ApoG2 binds to Bcl-2 and prevents its association with BH3-only pro-apoptotic proteins, allowing the pro-apoptotic proteins to participate in the execution of cell death.

When used as a single agent at 120 μmol/kg daily, ApoG2 exhibited *in vivo *cytoablative activity in Bcl-2-transgenic mice as measured by weight, size, and B-cell counts in spleen [37]. The Bcl-2-expressing B-cells from transgenic mice were more sensitive to cytotoxicity induced by ApoG2 than gossypol *in vitro *with LD_50 _values of 3 to 5 μM and 7.5 to 10 μM, respectively. Using the maximum tolerated dose (MTD) of gossypol for sequential daily dosing during in vivo studies, apogossypol displayed superior activity than gossypol in terms of reducing splenomegaly and reducing B-cell counts in spleens of Bcl-2-transgenic mice [37].

Additional studies from our laboratory have shown that ApoG2 has potent anti-lymphoma effect *in vitro *on the WSU-FSCCL cell line [38,39] exhibiting IC_50 _value which is 9- and 18-fold lower when compared to TW-37 and gossypol. TW-37 is a benzenesulfonyl derivative, which was designed to target the BH3-binding groove in Bcl-2 where proapoptotic Bcl-2 proteins, such as Bak, Bax, Bid, and Bim bind. Our laboratory has demonstrated the in vivo efficacy of TW-37 in WSU-DLCL_2_-SCID mouse xenografts with tumor growth inhibition (T/C) value of 28%, tumor growth delay (T-C) of 10 days and log_10_kill of 1.50. We have also shown that ApoG2 could significantly increase the life span of lymphoma-bearing SCID mice by at least 42%

Although another SMI viz. ABT-737 (discussed below) has a considerably lower IC_50 _(8 and 30 nM) when used against FL cell lines, it fails to bind to Mcl-1 posing a potential problem since Mcl-1 expression may inherently result in resistance. In comparison, ApoG2 targets all these three anti-apoptotic proteins. ApoG2 as a single agent has shown efficacy in treatment of FL and is likely to be even more effective when used in combination with standard chemotherapy.

#### B. ABT- 737

**Figure 3:**
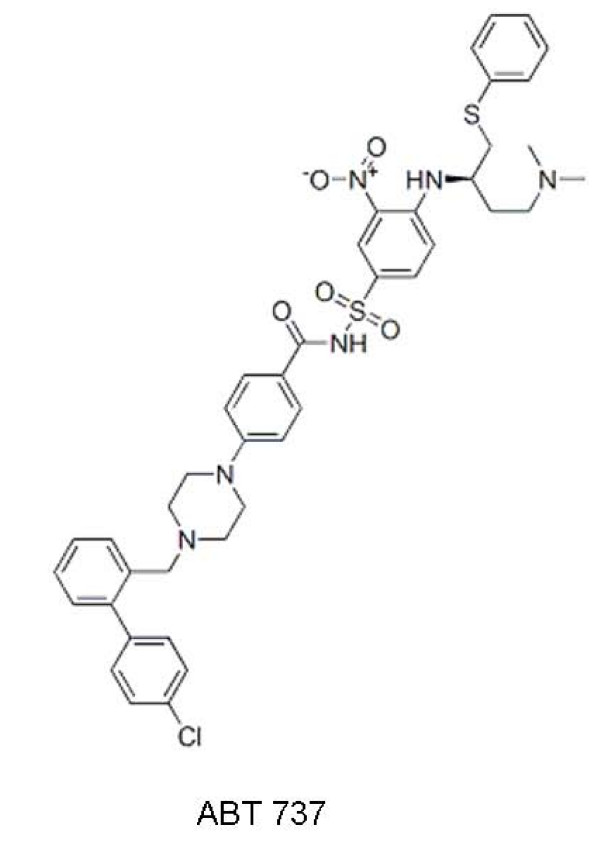
**Chemical structure of ABT-737**.

ABT-737 (Figure 3) was developed in collaboration between IDUN and Abbott laboratories. It has been shown to inhibit Bcl-X_L_, Bcl-2 and Bcl-W, but not Mcl-1, Bcl-B and A1 [40]. The inability of the drug to neutralize Mcl-1 may provide an explanation why certain tumors are resistant to ABT-737. Experiments have shown that down-regulation of Mcl-1 dramatically potentiates lethality of ABT-737 by releasing Bak from both Bcl-X_L _and Mcl-1 which results in simultaneous induction of Bak and Bax [41].

ABT-737 has demonstrated single agent efficacy against human FL cell lines that overexpress Bcl-2. The drug has also yielded very impressive results in a murine xenograft model of lymphoma when given both as a single agent and in combination with etoposide [42]. Mice tolerated daily injections for three weeks with no adverse effects except a decline in platelets and lymphocytes. When SCID mice implanted with a human FL cell line were treated with ABT-737, morbidity was noticeably delayed [42]. This drug is presently in phase II of clinical testing.

#### C. ABT- 263

**Figure 4:**
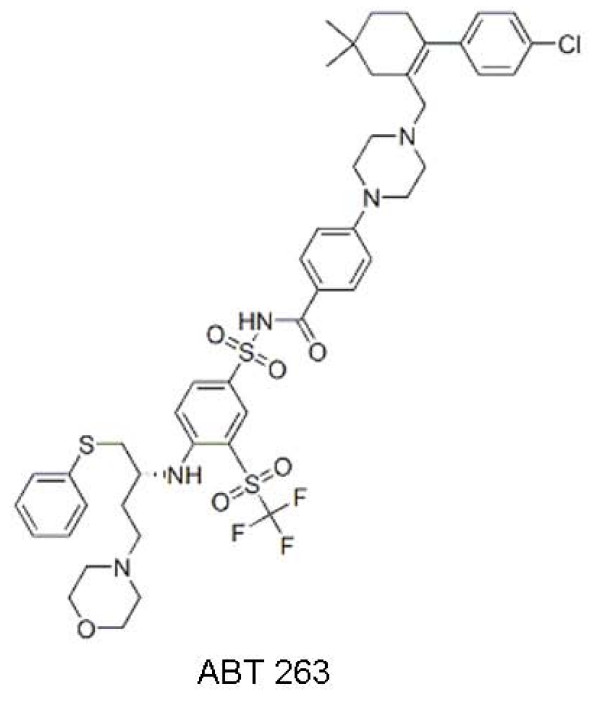
**Chemical structure of ABT-263**.

ABT-263 (Figure 4) is a potent orally bioavailable SMI that is structurally related to ABT-737. This Bad-like BH3 mimick disrupts Bcl-2: Bcl-X_L _interactions with pro-apoptotic proteins inducing cytochrome c release and subsequent apoptosis [43,44]. As with ABT-737, this agent does not possess a high affinity for Mcl-1 [45]. Oral administration of ABT-263 alone has previously been shown to induce complete tumor regressions in xenograft models of small-cell lung cancer and acute lymphoblastic leukaemia [46,47].

Recently, ABT-263 in combination with Rapamycin has shown significant efficacy in FL cell lines [48]. In xenograft models of these tumors, rapamycin induced a largely cytostatic response in the DoHH-2 and SuDHL-4 models. However, co-administration with ABT-263 induced significant tumor regression, with DoHH-2 and SuDHL-4 tumors showing 100% overall response rates.

The phase IIa portion of a multicenter study is evaluating ABT-263 in up to 40 subjects who have follicular and aggressive NHL to obtain a preliminary assessment of efficacy. The pharmacokinetic profile of ABT-263 has been shown to be linear between 10 mg and 160 mg/dose. The average terminal half-life of ABT-263 varied between 14 and 25 hours across all dose levels. It reduced the platelet levels in a dose-dependent manner [49]. No other major toxicity has been noted.

#### D. HA 14-1

**Figure 5:**
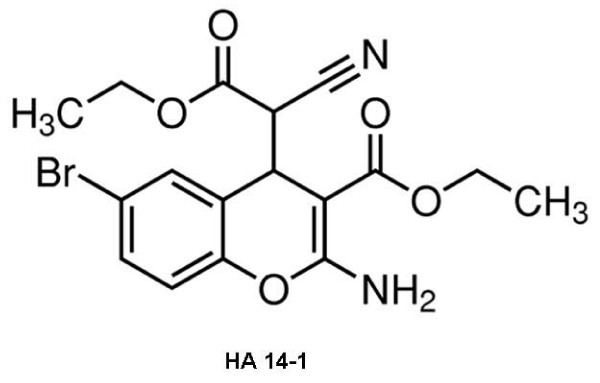
**Chemical structure of HA 14-1**.

HA 14-1 (Figure 5) was the first reported Bcl-2 binding molecule identified by using a computer-aided design strategy based on the predicted structure of Bcl-2 protein [50]. It binds to the surface pocket of Bcl-2 with high affinity, inhibiting the interaction with Bak, thereby triggering dissipation of mitochondrial membrane potential and activating caspases [51]

Skommer et al showed that HA 14-1 is a potent inducer of apoptosis in human FL cells [52]. Moreover, HA14-1 significantly enhanced dexamethasone- and doxorubicin-mediated, but not vincristine-mediated, cytotoxicity and apoptosis [53]. For this reason, use of HA14-1 may be an efficient strategy to lower the tumor response dose of doxorubicin, decreasing its cardiotoxicity and nephrotoxicity [53].

HA 14-1 has also shown an ability to enhance Brefeldin A (BFA) mediated cell killing in FL cell lines [54]. BFA-induced cell death is associated with profound ER stress, mitochondrial breach and subsequent caspase cascade activation with clear predominance of apoptosing cells at a G1 phase of the cell-cycle [53,54]. The apoptosis induced by HA 14-1 is cell-cycle specific, with the G1 and S phases of the cells being targeted frequently. Combining HA 14-1 with drugs acting on the G1 and/or S phase may potentially be of value. However, HA 14-1 is an unstable compound and decomposes very rapidly under physiological conditions. Due to its instability and redox activity, a newer and stable molecule, viz. sHA 14-1 has been developed, which has better *in vitro *activity against cancer cells [53,54].

## B) p53-MDM2 interaction inhibitors

The idea of creating a magical bullet that could help to unlock wild-type p53 and re-gain its functional activity in cancer cells is currently of interest and under experimental investigation. The tetrameric phosphoprotein p53 plays a central role in regulating the cell cycle in response to various kinds of stress, such as oxidation or radiation [55-58]. In normal cells, p53 is highly unstable with half-life measuring in minutes. However, the half-life increases significantly in response to cellular stress, leading to activation of multiple downstream genes implicated in apoptosis, senescence and cell cycle control. The p53 function has been found to be impaired in nearly 50% of cancers by either a mutation or deletion in the TP53 gene [59]. As a consequence, activated p53 is detrimental to the proliferation of cancer cells.

**Figure 6:**
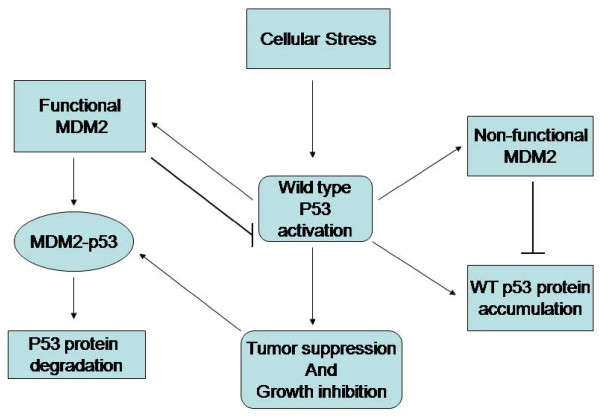
**A representative pathway of p53-MDM2 autoregulatory loop**.

MDM2 was initially found as a product of an oncogene amplified in a mouse tumor cell line [59-62]. In non-cancerous cells, it binds to p53 as a complex and promotes its degradation by ubiquitination [60]. Thus, deregulation of MDM2 could provide significant growth advantage. The MDM2 gene has been found to be over-expressed by amplification in several cancers with the highest frequency observed in soft tissue sarcomas. The primary function of MDM2 is to regulate p53 levels. These two molecules regulate each other through an autoregulatory feedback loop (Figure 6). When the levels of p53 are elevated, it transcribes the MDM2 gene, concurrently raising the level of its protein product. MDM2, leading to inactivation of p53 by either binding to the p53 transactivation domain or facilitating its degradation by exporting p53 out of the nucleus. MDM2 also acts as an E3 ubiquitin ligase targeting the p53 for degradation. Deletion of MDM2 gene in mice is lethal, but can only be reversed by simultaneous deletion of the TP53 gene [63,64]. In addition, genetically engineered mice expressing reduced levels of MDM2 are small in size, have reduced organ weight, and are radiosensitive [65], providing further evidence of this protein-protein interaction. Protein-protein interactions involve large and flat surfaces that are difficult to target by low molecular weight molecules. It is clear by now that p53-MDM2 interface showcases a unique and rather unusual protein-protein interaction [66]. The hydrophobic residues of Phe19, Trp23 and Leu26 project into a deep and highly structured pocket on the MDM2 surface, which can be targeted by a nonpeptide SMI, thus unlocking and reactivating p53.

### Small-Molecule Inhibitors of p53-MDM2 Interaction

**Figure 7:**
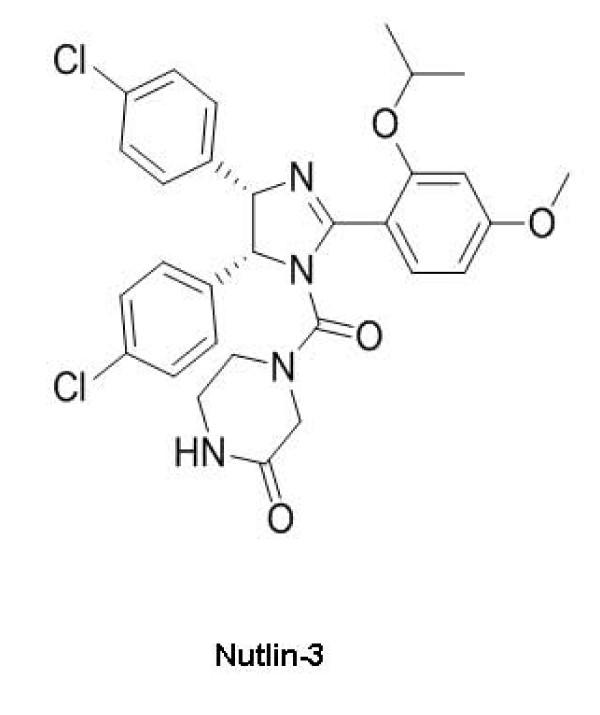
**Nutlins, newly designed Small-Molecule Inhibitors of p53-MDM2 interactions**.

In 2004, Vassilev et al described a class of antagonists that targeted the p53-MDM2 interaction [67]. Identified from a group of *cis*-imidazoline compounds, these were designated as Nutlins (see Figure 7). Based on crystallographic studies nutlins have been shown to interact with the hydrophobic cleft of MDM2, thus mimicking the binding of the helical portion of p53. However, one of the enantiomers of this racemic mixture of compounds was found to possess higher affinity for the binding site as compared to others. The active enantiomers of the *cis*-imidazoline analogues were named Nutlin- 1, -2 and -3.

The investigators showed that incubation of wild-type p53 cancer cells with Nutlins for eight hours led to a dose-dependent increase in the cellular levels of p53, MDM2 and p21. At 24 hours post-treatment, a significant G1/M phase fraction was observed with depletion of S phase suggesting cell cycle arrest. This alteration was not observed in cell lines with mutant or deleted p53 cancer cell lines. Only cells with wild-type p53 respond to these SMIs. Nutlin- 3a was administered for three weeks to nude mice bearing human cancer xenografts, which led to effective tumor inhibition and shrinkage.

**Figure 8:**
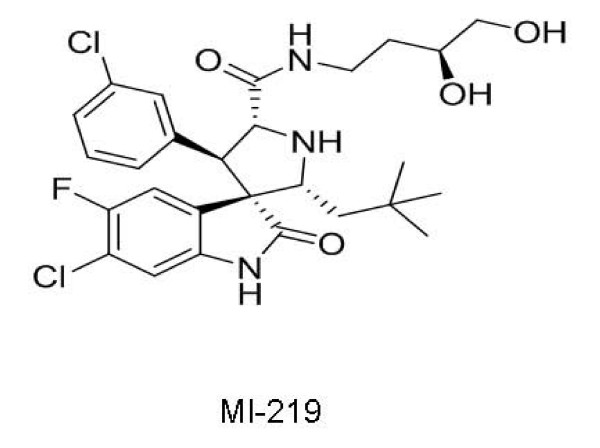
**Structure of MI-219, a MDM2 inhibitor**.

Ding et al at the University of Michigan have identified compounds with spiro-oxindole core structure as a new class of SMIs targeting p53-MDM2 interaction [68]. Treatment with MI-219 (Figure 8) induced p53 accumulation and up-regulation of MDM2, p21, and PUMA, three p53-target gene products, in SJSA-1 (osteosarcoma), LNCaP and 22Rv1 (prostate cancer) cell lines with wild-type p53 in dose dependant manner [69].

However, restoring p53 activity in tumor cells could also trigger p53 in normal tissues leading to deleterious consequences. A genetic study showed that mice with 70% reduced MDM2 expression developed normally but had reduced body weight and mild disturbance in hematopoiesis with increased apoptosis in small intestine [70]. On the other end of the spectrum, a study showed that p53 was spontaneously active in all tissues of MDM2 deficient mice, causing severe toxicity and leading to rapid animal death [71]. In comparison, activation of p53 by MI-219 is always under the surveillance of MDM2 and is therefore never fully out of control [69].

**Figure 9:**
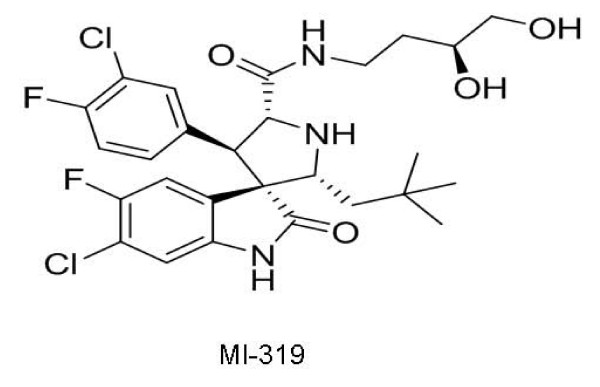
**Chemical structure of MI-319**.

In our lab MI-319 (Figure 9), which is close analogue of MI-219, had shown potent anti-lymphoma activity against the WSU-FSCCL cell line *in vitro *and *in vivo*. Both the compounds displayed comparable binding affinity for the MDM2 protein in our fluorescence polarization-based competitive binding assay. In the xenograft model that was established by injecting 2 × 10^7 ^WSU-FSCCL cells per mouse, treatment with MI-319 showed a significant therapeutic impact (article in press).

## C) Proteasome Inhibitors

The ubiquitin-proteasome pathway plays a key role in the degradation of misfolded or unwanted intracellular proteins in eukaryotic cells [72,73]. Despite help from chaperones, more than 80% of proteins fold incorrectly. Poly-ubiquitination of these proteins targets them for degradation by the 26S proteasome, a highly conserved multi-protein complex [74]. This ATP dependent multi-catalytic protease unit is present in numerous copies throughout the cytosol and the nucleus. The 26S proteasome is composed of a catalytic 20S core with four heptameric rings of alpha and beta subunits stacked into a hollow cylinder [75,76]. Two 19S subunits, containing proteasome activators that recognize tagged proteins for degradation, are found at the end of this cylinder.

Some of the proteins targeted by this complex include p53, p21, p27, the inhibitory protein (I-.B), and Bcl-2 respectively [77]. Preclinical studies have shown that inhibition of this pathway can lead to inhibition of tumor metastasis, angiogenesis and induction of cell death. Furthermore, malignant cells are much more sensitive to the effects of proteasome inhibition than normal cells [78,79].

The ubiquitin-proteasome pathway is a key mechanism in deciding the activity of cell-cycle regulatory proteins. Inactivation of mitotic cyclin dependent kinases (CDKs) by proteolytic destruction of B-type cyclins was the first cell-cycle regulatory event shown to be mediated by a ubiquitin-dependent proteasomal pathway [80-82]. The ordered degradation of p21 and p27 is required for progression through cell-cycle and mitosis. Uncontrolled activity of p21 and p27 can cause cell-cycle arrest by inhibition of CDK. It is now known that the SCF family of ubiquitin-protein ligases is responsible for protein ubiquitinylation in the G1/S phase and the related APC/cyclosome complexes perform the same function in G2/M. We are only beginning to understand the extent to which deregulation of cell-cycle regulators contributes to human cancer.

In addition, the ubiquitin-proteasome system plays a critical role in the degradation of IK-kB, an intracellular protein that acts as a negative regulator of nuclear factor kappaB (NF-B) [83-85]. NF-.B is responsible for the activation of several genes that promote cell proliferation, cytokine release, anti-apoptosis, and changes in cell surface adhesion molecules. NF-B is sequestered in the cytoplasm when complexed with IK-B, and cannot enter the nucleus to promote transcriptions of all its target genes. Hence, stabilization of IB through proteasome inhibition would prevents NF-B activation, making cells more susceptible to environmental stress and cytotoxic agents. The overexpression of the pro-survival protein Bcl-2 in follicular lymphoma due to the translocation of the gene t(14;18)(q32;q21) can be mediated through the inhibition of the 26S proteasome, which could make FL cells particularly vulnerable to inhibitors of this pathway.

### Bortezomib in Follicular Lymphoma

**Figure 10:**
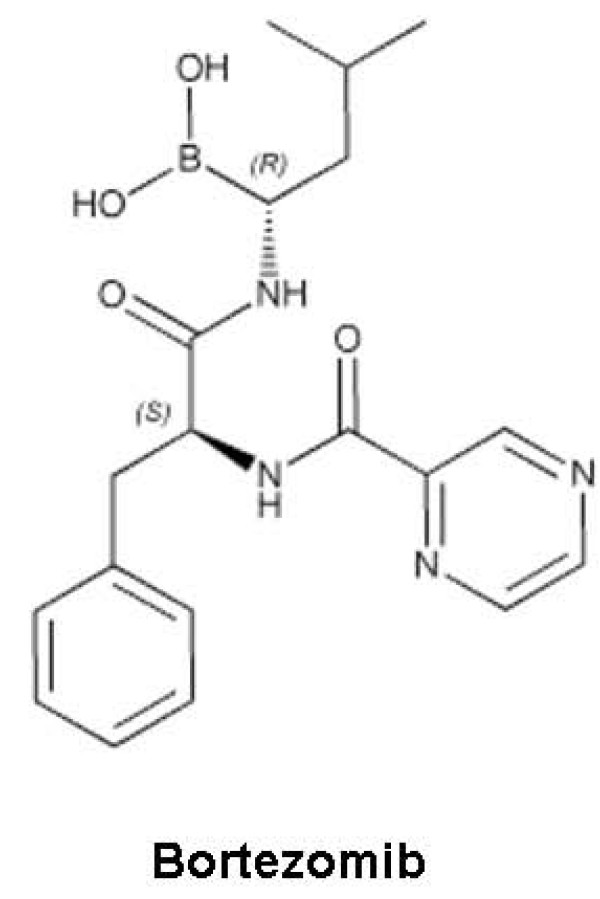
**Structure of a new clinically approved proteasome inhibitor, Bortezomib**.

Bortezomib (Velcade, Millenium Pharmaceuticals) (Figure 10)was the first member of a new class of proteasome inhibitors to be evaluated in human trials. It has been approved by FDA for treatment of patients with multiple myeloma, from diagnosis till relapse and beyond. Pre-clinical studies have demonstrated encouraging results with this proteasome inhibitor in NHL cell lines [84]. It has been shown to induce apoptosis in primary effusion lymphoma (PEL) cell lines through upregulation of p21, p27 and p53 [86,87]. It was shown to be effective in inhibiting cells from both FL and MCL patients with the median IC_50 _being significantly lower for MCL [88]. This drug was further shown to prevent tumor growth in MCL-xenografted mice [89]. More encouraging results have been seen with combination therapy involving bortezomib. It has been shown that synergistic effect with bortezomib is even greater if cells are sequentially treated with vincristine or doxorubicin and then bortezomib [90]. Pre-treatment with bortezomib has also been found to be more beneficial when used in combination with paclitaxel or doxorubicin in PEL cell lines [87].

Several Phase II studies subsequently undertaken in the past few years have established the efficacy of this novel drug in various subtypes of NHL. In 2006, FDA approved the use of bortezomib in patients with mantle cell lymphoma (MCL) who have received at least one chemotherapy regimen, based on the findings of the PINNACLE trial [91]. This prospective, multi-center, single-arm, open-label study was undertaken in patients with MCL whose disease progressed following at least one prior therapy. Overall response rate was 31% with complete response (CR + CRu) rate of 8 percent. The median duration of response of 9.3 months and 15.4 months in patients were achieving a CR.

Preliminary data from several ongoing studies indicates that bortezomib is an effective agent in FL with some durable overall responses (ORRs) of 18-60%. In an NCI-sponsored phase 2 study, bortezomib was given to patients with relapsed indolent NHL on the conventional schedule of twice weekly for 2 out of 3 weeks (1.5 mg/m2) [92]. The ORR in 19 patients with FL was 60% with 1 CR, 1 Cru and 7 PR. Another phase II study in patients with relapsed or refractory B-cell NHL reflected one possible Cru out of 5 patients with FL [93]. A third study by Strauss et al used bortezomib at 1.3 mg/m2 with conventional schedule and showed that 2 out of 11 evaluable patients achieved a PR for an ORR of 18% three months after treatment [94]. As compared to the previous study with greater response rates, treatment was discontinued in non-responders, even without progression.

It has been suggested that the time to response in FL may be longer than other lymphomas due to its indolent course, suggesting a need for prolonged treatment. Optimizing the dosing and the schedules will also be a challenge given the biological heterogeneity of FL and the varying synergistic interactions with other SMIs.

## D) TRAIL activators

Another successful effort in developing selective SMIs for cancer therapy has been targeting death receptors on the extra-cellular membrane. TRAIL is expressed constitutively on a subset of natural killer (NK) cells in liver and may be induced on monocytes, dendritic cells, B-cells and T-cells by signal from TLRs or interferons. Five receptors for TRAIL have been identified, two of which, death receptor DR4 (TRAIL-R1) and DR5 (TRAIL-R2), are capable of transducing the apoptosis signal.

After binding of either the ligand or agonist antibody to the extracellular domain of TRAIL-R1, a death-inducing signaling complex (DISC) that includes Fas-associating protein is formed with FADD and caspase-8 or -10 [95]. Once activated, this cascade of caspases degrades critical regulatory proteins and DNA, resulting in the characteristic morphology of PCD [96]. Expression of DR4 & -5 is frequently detected in human cancers including colon, gastric, pancreatic, ovarian, breast and non-small-cell lung cancer, with low or no expression in normal tissues [97]. Zerafa et al demonstrated the role of TRAIL as a tumor suppressor in mice that are mutant for one p53 allele. TRAIL deficiency predisposed mice to a greater number of tumors, including disseminated lymphomas and sarcomas [98]. In fact, greater than 25% mice developed lymphoid malignancies after 500 days of life.

Triggering the TRAIL receptor could be an effective means of targeting cancer cells with inactivated p53 mutations because death-receptor mediated cell death is independent of p53. In this effort, agonistic antibodies to DR4 and DR5 have been generated. Recently, a mouse agonistic antibody against DR5, TRA-8, has been shown to have strong tumoricidal activity *in vivo *[99]. It has shown to be very effective in human breast cancer xenograft model [100]. These new class of antibodies are moving at a swift pace from benchside to the clinic.

### TRAIL in Follicular Lymphoma

To establish if TRAIL could be a potential therapeutic target in FL, Travert et al estimated its potency to induce apoptosis on B-cells from FL patients [101]. After a 24 hour treatment with 500 ng/ml TRAIL on cells extracted from lymph nodes recovered from patients with FL at diagnosis, the percentage of active caspase 3-positive cells on CD19^+^CD20^+ ^B lymphocytes were estimated by flow cytometry. All the patients (n = 11) were found to be sensitive to TRAIL. A 30% increase of active caspase 3-positive primary FL B-cells according to the control was noted. Interestingly, an average 20% of active caspase 3-positive non-treated cells were detected reflecting spontaneous apoptosis after 24 hours of culture, thus underlining the potential role of tumor micro-environment in the pathogenesis of FL.

On the other hand, a phase I study with the agonistic antibody Mapatumumab showed that this molecule has no significant hematological toxicity [102]. Similarly, a phase II trial targeting DR4 in patients with relapsed/refractory NHL has reported an objective response in 14 patients with FL, including one CR [103]. It is becoming clear that one critical determinant of response is the selection of optimal patients and chemotherapy regimens to be combined with TRAIL receptor-targeting agents. Examination of drug resistant FL cell lines has revealed that mutations that inhibit the upregulation of p53 or expression of caspase-3 in the TRAIL pathway severely affect the ability of DNA-damaging drugs to circumvent the anti-apoptotic Bcl-2 block in FL [104]. Additional studies show that mutational inactivation of Bax and overexpression of Bcl-2 cause resistance to death receptor mediated apoptosis [104]. It can thus be foreseen that using agents that restore p53 function (such as the MDM2 inhibitors) or immunological agents like Rituximab in pairing with agonistic TRAIL antibody could enhance responses to standard chemotherapy agents by overcoming tumor cell resistance.

## E) Thymoquinone as an apoptosis inducing agent for follicular lymphoma

**Figure 11:**
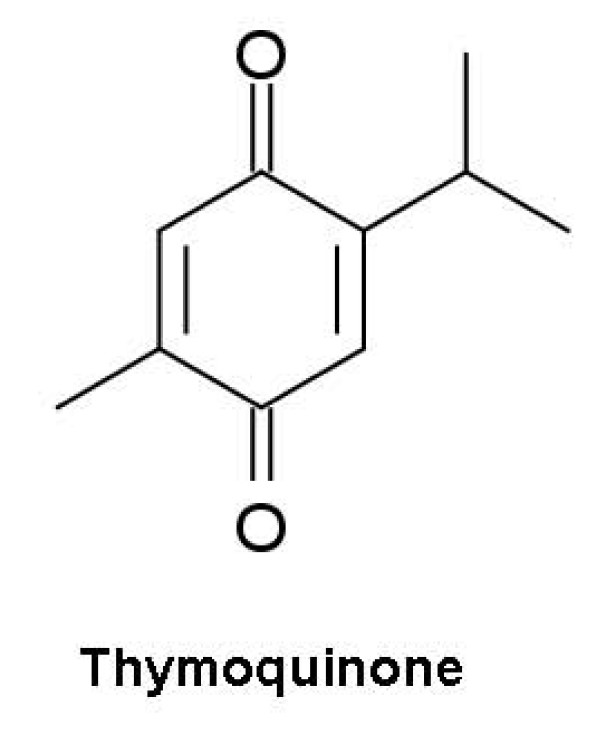
**Structure of Thymoquinone extracted from Nigella sativa seed**.

Thymoquinone (TQ) (Figure 11) is an active constituent of volatile oil of black *Nigella sativa *seed with biological activities that we have detailed in our recent report [105]. TQ has good safety profile with LD50 value of 104.7 and 57.5 mg/kg after i.p. injection and 870.3 and 794.3 mg/kg after oral treatment in mice and rats respectively [106]. Despite its impressive safety profile and potent anticancer activity, there are no reports available in the literature about use of TQ in the treatment of FL. We have performed limited in vitro studies using a WSU-FSCCL cell line and found that TQ can inhibit up to 50% cell growth by using 3 micro-molar concentrations. In this review we provide rationale to explore the use of TQ for the treatment of FL.

The anti-proliferative effect of TQ has been studied in cancer and normal cell lines, viz. canine osteocarcinoma (COS31) and its cisplatin-resistant variant (COS31/rCDDP), human breast adenocarcinoma (MCF-7), Human ovarian adenocarcinoma (BG-1) and Mandin-Darby canine (MDCK) cells respectively [107]. The cell cycle checkpoints allow the cells to correct possible defects and avoid progression to cancer [108,109]. There are two major checkpoints to identify DNA damage: one at the G1-S transition which prevents the replication of damaged DNA and other at the G2-M transition that prevents non-intact chromosome segregation. The apoptosis inducing activity of TQ was found to be due to its effects on the expression of cell cycle regulatory proteins. TQ inhibit G1 phase of cell cycle via increase in the expression of the cyclin-dependent kinase inhibitor p16 and downregulation of cyclin D1 protein expression in papilloma cells [110]. Treatment with TQ in HCT-116 cells has been found to lead to G1 arrest associated with up-regulation of p21^WAF1 ^cells which blocks CDK2 activity and possibly CDK4 and CDK6 activities which were suggested the principal transcriptional target of p53 in the context of the G1 checkpoint [111]. TQ was also found to arrest G2/M phase of cell cycle which was associated with an increase in p53 expression and down-regulation of cyclin B1 protein in spindle carcinoma cells. TQ induced apoptosis was mediated via p53 which can regulate G2/M transition through either induction of p21 or 14-3-3sigma, a protein that normally sequesters cyclin B1-CDC2 complexes in the cytoplasm [112-115]. Antiproliferative and pro-apoptotic effects of TQ are mediated by induction of p53-dependent apoptosis in human colon cancer cells which is supported with a study by Roepke and colleagues [116] in two human osteosarcoma cell lines with different p53 mutation status using flow cytometry and DNA damage assays. TQ induced a much larger increase in the Pre-G1 (apoptotic) cell population, but no cell cycle arrest in MG63 cells, in the flow cytometric analysis, on other hand TQ was confirmed to induce greater extent of apoptosis in p53 null MG63 cells by using three DNA damage assays. The upregulation of p21^WAF1 ^was associated with G2/M arrest in MNNG/HOS cells. Both cell lines did not show any modulation of Bax/Bcl-2 ratios. The apoptosis induced by TQ showed involvement of the mitochondrial pathway due to cleavage of caspases-9 and -3 in MG63 cells. TQ triggers apoptosis in a dose and time-dependent manner, starting at a concentration of 100 μM after 12 h of incubation which is associated with a 2.5 to 4.5 fold increase in p53 and p21^WAF1 ^mRNA expression and a significant decrease in Bcl-2 protein levels in HCT-116 cells. Co-incubation with pifithrin-α, a p53 inhibitor, restored the Bcl-2, p53 and p21^WAF1 ^levels to the untreated control levels and absolved the effects of TQ [117].

Altogether, these results suggest that TQ is involved in influencing cell cycle regulators involved in apoptosis as well as in down-regulation of the anti-apoptotic proteins, which is supported by similar effects on primary mouse keratinocytes, papilloma (SP-1) and spindle carcinoma cells respectively. At longer incubation times (48 h) the compound induced apoptosis in both cell lines by increasing the ratio of Bax/Bcl-2 protein expression and down-regulating the Bcl-xL protein [118]. TQ has been shown to initiate apoptosis even via p53-independent pathways through activation of caspase-3, 8 and 9 in p53-null myeloblastic leukemia HL-60 cells [119]. It was observed that caspase-8 activity was highest after 1 h following the treatment of TQ, while caspase-3 activity was highest after 6 h respectively. These observations were explained on the basis of up-regulation of pro-apoptotic Bax protein along with down-regulation of antiapoptotic Bcl-2 proteins resulting in enhanced Bax/Bcl-2 ratio. These results are also supported by reports in prostate and other cancer cells [120-122].

Recently we found that TQ is very effective against FL, DLCL and Hodgkin's *in vitro*. Usually, the IC50 of TQ against cancer is high, but our recent data showed that the IC50s for TQ against WSU-FSCCL, WSU-DLCL2 (non-Hodgkin's) and KM-H2 (Hodgkin's) are between 1-3 μM, which makes TQ a very important dietary supplement in lymphoma. In addition, TQ combination with standard chemotherapeutic regimen such as CHOP or R-CHOP (rituxin, cyclophosphamide, doxorubicin, vincristine and prednisone) showed a better antilymphoma efficacy.

Recent studies on TQ have suggested that NF-kB is a legitimate target of its action in cell growth inhibition and induction of apoptosis in cancer cells. TQ showed down-regulation of gene products of NF-kB-regulated antiapoptotic proteins (IAP1, IAP2, XIAP Bcl-2, Bcl-xL, and survivin), proliferative (cyclin D1, cyclooxygenase-2, and c-Myc), and angiogenic factors (matrix metalloproteinase-9 and vascular endothelial growth factor) [123]. TQ also showed dose- and time-dependent reduction of PDA cell synthesis of MCP-1, TNF-alpha, interleukin (IL)-1beta and COX-2, while after 24 h treatment it completely abolishes inflammatory mediators in pancreatic cell line [124]. In our previous study, we found out that TQ could potentiate the killing of pancreatic cancer cells induced by chemotherapeutic agents like gemcitabine or oxaliplatin by down-regulation of nuclear factor-kappaB (NF-kappaB), Bcl-2 family, and NF-kappaB-dependent antiapoptotic genes (X-linked inhibitors of apoptosis, survivin, and cyclooxygenase-2) [125]. TQ also showed antiangiogenic activity *in vitro *and *in vivo *in a xenograft human prostate cancer (PC3) in mouse [126]. A recent report has identified checkpoint kinase 1 homolog, CHEK1, a serine/threonine kinase, as the target of TQ, leading to apoptosis in p53+/+ colon cancer cells. The study compared the effect of TQ on p53+/+ as well as p53-/- HCT116 colon cancer cells where the former were found to be more sensitive to TQ in terms of DNA damage and apoptosis-induction. As a possible explanation for such sensitivity, it was observed that CHEK1 was up-regulated upto 9 folds in p53-null HCT116 cells. Further, transfection of p53 cDNA and CHEK1 siRNA in p53 null cells resulted in restoration of apoptosis to the levels of p53+/+ cells. The *in vivo *results demonstrated that tumors lacking p53 had higher levels of CHEK1 which was associated with poorer apoptosis, advance tumor stages and worse prognosis [127].

Thus, there is compelling evidence that TQ induces apoptosis through modulation of multiple targets and hence constitutes as a promising phytochemical for initiation of many types of cancer cells. As discussed in this review, targeting Bcl-2, p53 and proteasome proteins for inducing apoptosis is emerging as an efficient strategy for treatment of FL. Since TQ has apoptosis inducing potential involving cell cycle arrest and upregulation of p53 followed by downregulation of NF-kB, bcl-2 and activation of Caspase-3,-9 pathways thus it is becoming increasingly clear that it offers a new treatment option for FL.

## Conclusion

Despite the impressive biological and therapeutic progress made in dealing with FL over the last decade, and a constantly growing number of FL patients being offered more hope for the disease-free survival time, there is still a substantial room for improving treatment. Tumor transformation into a more aggressive phenotype and development of resistance to standard chemotherapy regimens in the course of FL remain the main causes of deaths in patients with this type of lymphoma. A large number of novel agents potentially useful in FL patients are in the clinical trial pipeline which includes new chemotherapeutics, *bcl2 *SMIs, monoclonal antibodies, apoptosis-inducing agents, and immunomodulators. These therapies could help to extend the duration of remission without adding any further burden of toxicity. It is also becoming clear that the therapy for FL also needs to be adapted to the patient's individual status, depending on the aggressiveness of the disease, gene-signatures and tumor microenvironment while relying on a continuously growing repertoire of salvage therapies.

## Competing interests

The authors declare that they have no competing interests.

## Authors' contributions

All the authors contributed equally in drafting the manuscript and revising it critically for important intellectual content. All authors have read and approved the final manuscript.
